# The Unfolded Protein Response at the Tumor-Immune Interface

**DOI:** 10.3389/fimmu.2022.823157

**Published:** 2022-02-14

**Authors:** Maurizio Zanetti, Su Xian, Magalie Dosset, Hannah Carter

**Affiliations:** ^1^ The Laboratory of Immunology, Department of Medicine and Moores Cancer Center, University of California San Diego, La Jolla, CA, United States; ^2^ Division of Medical Genetics, Department of Medicine, Bioinformatics and System Biology Program, University of California San Diego, La Jolla, CA, United States

**Keywords:** unfolded protein response (UPR), myeloid cells, T cells, aneuploidy, transcellular stress

## Abstract

The tumor-immune interface has surged to primary relevance in an effort to understand the hurdles facing immune surveillance and cancer immunotherapy. Reports over the past decades have indicated a role for the unfolded protein response (UPR) in modulating not only tumor cell fitness and drug resistance, but also local immunity, with emphasis on the phenotype and altered function of immune cells such as myeloid cells and T cells. Emerging evidence also suggests that aneuploidy correlates with local immune dysregulation. Recently, we reported that the UPR serves as a link between aneuploidy and immune cell dysregulation in a cell nonautonomous way. These new findings add considerable complexity to the organization of the tumor microenvironment (TME) and the origin of its altered function. In this review, we summarize these data and also discuss the role of aneuploidy as a negative regulator of local immunity.

## Introduction

The tumor-immune interface is a variable of fundamental relevance to understand what determines tumor evolution. For a long time the relation between the immune system was considered immune-centric, meaning the entire focus was on the immune system as a protective measure. This helped formulate the immune surveillance (1, 2) theory with antigen on tumor cells as its cornerstone. Burnet defined the problem in a simple way: “*Any chemical configuration that is not genetically proper to the body can provoke a specific immune response. It is axiomatic to scholars interested in the general pathology of cancer that in most or all cancers the cell membrane differs from normal state. If these changes are due to somatic mutation, they probably represent either a new peptide configuration or some protein that is different enough from any normal self-pattern to be potentially antigenic*” (3). This concept served as the nexus to a tumor-centric view where considerations of individuality have taken the center stage due in great part to the opportunities offered by genomic analysis of cancers (4). This has lent support to the concept of tumor escape from control by the immune system, which became the new focus in revisions of the immune surveillance theory renamed immunoediting theory (5, 6). A tumor fulfills the biological necessity of surviving using conserved mechanisms such as de-repression of telomerase (7–9) and immune evasion. The tumor-immune interface marks a more recent interest driven in part to a better understanding of the tumor microenvironment and its relation with the mutational landscape (10), both motivated by the need to improve the success of immunotherapy (11). Hence, the proposal to fragment the tumor microenvironment in subclasses of immune environments (11).

We believe that beside enormous molecular specificity and mutational diversity in tumor cells and a parallel diversification and selection of receptors on immune cells, there exists a more primitive form of regulation one could equate to a *community effect*, a phenomenon based on cell-to-cell cooperation at play in animal development (12) also proposed to serve as a regulatory element of tumor evolution (13, 14). A somewhat analogous system exists in bacteria under the term of *quorum sensing* (15, 16), a mechanism through which bacteria detect fluctuations in extracellular cues and transduce sensory information into the cell to modulate gene expression in response to a changing environment that leads to the production and release of chemical messengers. Likewise, a *community effect* type of cell-cell communication has been reported in plants (17). And in slime mold, a single-celled soil-dwelling amoeba, cAMP released by a few cells causes the more numerous receiver cells to behave like transmitting cells during aggregation (18). Taken together, these considerations support the view that cell-cell cooperation is a fundamental phenomenon of evolution and it applies to cancer (19, 20).

Our view and the thesis of this review article is that the tumor-immune interface is a point of encounter and collision among cells with different origin and trajectories that needs to conform to a pattern conserved enough to make evolutionary sense. We and others have identified the response to stress of the endoplasmic reticulum (ER) or unfolded protein response (UPR) as one such mechanism. This review article will focus primarily on the interactions between tumor cells on the one hand, and myeloid cells (macrophages and dendritic cells) and T cells on the other. We elected to concentrate on these cell populations for there exists which more experimental evidence. We will discuss the implication of this signaling system in modulating the tumor microenvironment, promoting tumor cell fitness and drug resistance, and dysregulating immune cells. We will also discuss recent data showing that aneuploidy is the source of UPR in cancer cells and this can lead to cell-nonautonomous dysregulation of immune cells, T cells and macrophages.

## The UPR

The UPR is a conserved physiological mechanism that permits normal cells to cope with changes of the environment as they impinge upon the homeostatic state of the cell. This molecular signaling mechanism is conserved among yeast, fungi, worm, fly, corals, and vertebrate and mammalian cells even though mammalian cells are able to cope with ER stress in a more sophisticated manner (21–24). This is not surprising since a recent analysis of the proteome landscape of the kingdoms of life showed, remarkably, that a high fraction of the total proteome mass in all kingdoms is dedicated to protein homeostasis and folding (25). Is it also an important mechanism applicable to cancer in general?

**Figure 1:**
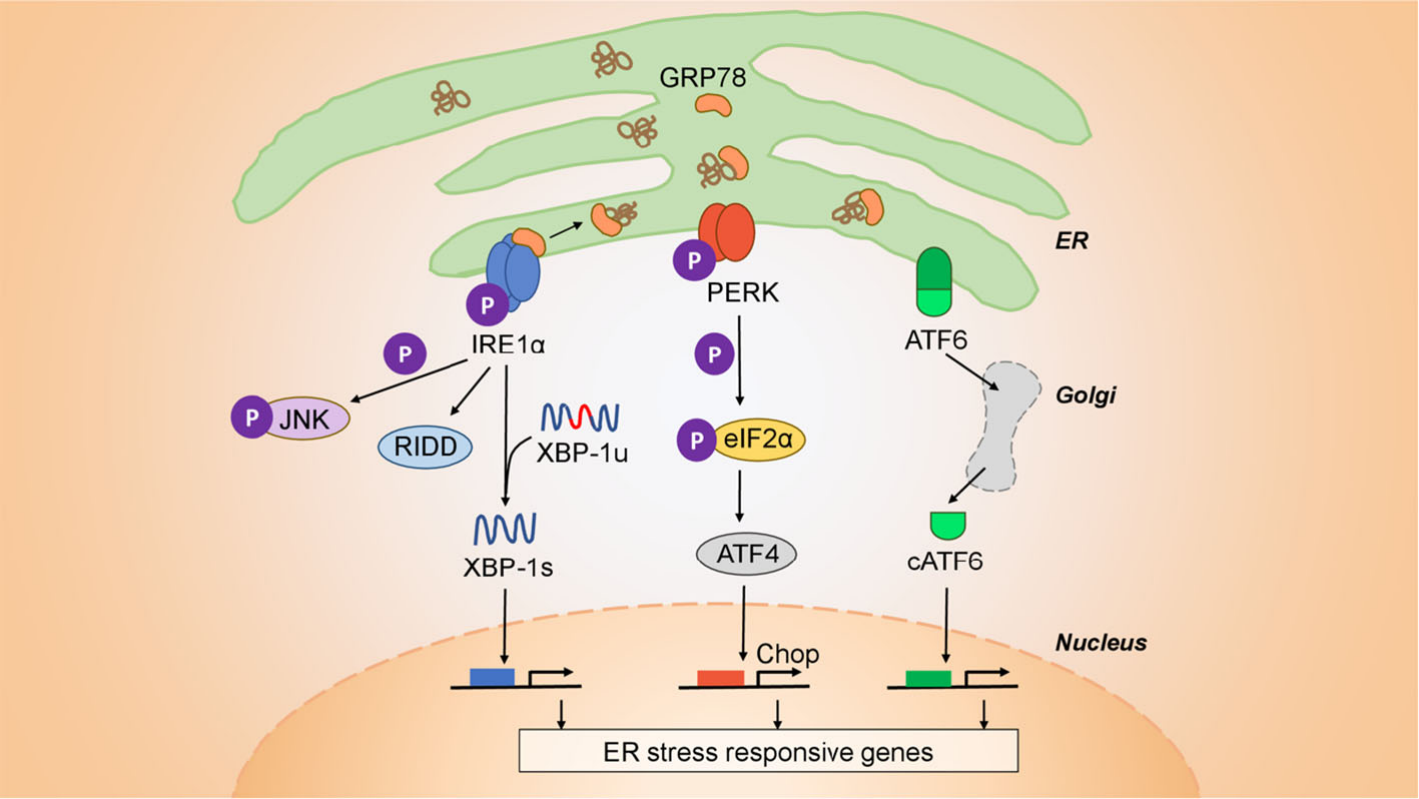
Architecture of the UPR. The UPR is mediated by three initiator/sensor molecules: inositol-requiring enzyme 1 (IRE1α), PKR-like ER kinase (PERK), and activating transcription factor 6 (ATF6). These three initiator/sensor molecules are maintained in an inactive state through association with 78 kDa glucose-regulated protein (GRP78) (29). Upon ER stress induction, PERK phosphorylates the eukaryotic translation initiation factor 2α (eIF2α) to control translation and further signal through downstream effectors such as the C/EBP homologous protein (CHOP) that modulates apoptosis. ATF6 translocates to the Golgi where it is cleaved into its functional form, and acts to restore ER homeostasis (32). IRE1α is an endoribonuclease that upon activation initiates the unconventional splicing of the mRNA encoding X-box-binding protein 1 (XBP1). Spliced XBP1 (XBP1s) is a potent transcriptional activator that increases expression of a subset of UPR-related genes involved in efficient protein folding, maturation, and degradation in the ER (30). IRE1α also has a kinase function independent of its endonuclease activity through which it signals downstream through c-JUN and TRAF2. Finally, IRE1α has an additional function that is independent of XBP1, is the endonucleolytic decay of many ER-localized mRNAs (regulated IRE1-dependent decay (RIDD).

Mammalian cells, and cancer cells in particular, are constantly subject to a variety of stressors including replication stress, DNA damage, heat shock, the integrated stress response, the unfolded protein response (UPR), and mitochondrial UPR (26). Cancer cells adapt to cell-extrinsic, environmental stressors (nutrient starvation, hypoxia, and acidic pH) and cell-intrinsic stressors (viruses, protein misfolding, and aneuploidy), to heighten their survival through the UPR. This signaling system is under the control of three initiator/sensor molecules, inositol-requiring enzyme 1 (IRE1α), PKR-like ER kinase (PERK), and activating transcription factor 6 (ATF6) (27, 28). Each consists of an ER luminal domain, a single transmembrane domain, and a cytosolic domain. This organization enables sensing misfolded proteins within the ER lumen and translates the signals to the cytosol to activate different downstream molecules. In the unstressed state, these sensor molecules are maintained inactive through association with the 78 kDa glucose-regulated protein (GRP78) (29). Upon ER stress induction, GRP78 disassociates from the three sensors, derepressing them and allowing downstream signaling to initiate. Both IRE1α and PERK contain kinase domains that autophosphorylate in trans. Activated PERK phosphorylates the eukaryotic translation initiation factor 2α (eIF2α), which acts as a negative regulator of translation to limit the number of client proteins in the ER. Unresolvable ER stress, however, activates downstream effector molecules ATF4 and the C/EBP homologous protein (CHOP) to induce apoptosis. The cytosolic portion of IRE1α contains both kinase active sites and the RNase domains. Upon activation IRE1α endoribonuclease initiates the unconventional splicing of the mRNA encoding X-box-binding protein 1 (XBP1). Spliced XBP1 is a potent transcriptional activator that increases expression of a subset of UPR-related genes involved in efficient protein folding, maturation, and degradation in the endoplasmic reticulum (30). An additional function of IRE1α independent of XBP1 is the endonucleolytic decay of many ER-localized mRNAs and miRNAs through a phenomenon termed regulated IRE1α-dependent decay (RIDD) (31). Upon activation, ATF6 translocates to the Golgi where it is cleaved into its functional form and acts in parallel with XBP1s to restore ER homeostasis (32). During unresolvable ER stress compensatory mechanisms fail and downstream signaling from PERK, *via* ATF4 and CHOP, initiates apoptosis (27). The ensemble of these signaling pathways is shown in **Figure 1**.

Evidence implicates ER stress and the UPR in tumorigenesis, cancer growth, and progression (33, 34). However, the role of the UPR in cancer can be further distinguished into cell-intrinsic, through which cells acquire greater fitness and pro-survival, and cell-extrinsic, which is mediated by soluble messenger molecules released by cancer cells undergoing a UPR that coopt receiver cells (35).

Examples of cell-intrinsic effects of the UPR on tumor cells abound. For instance, breast cancer possesses high levels of GRP78 (36). In these conditions of GRP78 elevated expression, translocation to the cell surface can also occur to serve as a signaling molecule to activate the phosphoinositide-3-kinase (PI3K) (37, 38) and promote growth and resistance to chemotherapy [for review see (39)]. XBP1 is activated in triple negative breast cancers (TNBC) and plays a pivotal role in tumorigenicity and cancer progression in humans (40). In mice, the conditional homozygous knockout of *Grp78* in the prostate of mice with *Pten* inactivation protects against cancer growth (41). The inactivation of PERK or a dominant-negative PERK in tumor cells, results in tumors that are smaller and less aggressive than their normal counterparts when implanted into mice (42). Inactivation of PERK and IRE1α results in impaired tumor cell survival under hypoxic conditions *in vitro*, and decreased tumor growth *in vivo* (42, 43). Thus, a tumor UPR initiates a cell-intrinsic signaling program that promotes tumor cell adaptation to the microenvironment resulting in enhanced survival and proliferation. However, a cell-intrinsic UPR is not the only manifestation of the UPR in cancer cells. An aspect of increasing interest is transcellular UPR and its effects not only on neighboring tumor cells (44) but also from tumor cells to immune cells.

## Transcellular UPR Transmission as a Regulatory Mechanism in the Tumor Microenvironment

Immunity has long been regarded as the guardian of tumor development and growth. However, what type of correlate distinguishes good vs. poor outcome remains a challenge. Abundance, density, and quality of T lymphocytes in tumors correlate with clinical outcome in many cancer types (36, 45–47). Notwithstanding the fact that only a small fraction (<10%) of intra-tumor T cells are specific for autologous tumor antigens (48), the ongoing anti-tumor response may require stem-like CD8 T cells in tumor-draining lymph nodes (49), and the response to immunotherapy may require pre-existing CD8 T cells at the invasive tumor margin (50). However difficult this might be, the potential benefit of tumor-infiltrating T cells is opposed by T suppressor/regulatory (Treg) cells (51) and by leukocyte infiltrates - most notably myeloid cells. Myeloid cells in the TME are of central relevance to understand the dynamics of tumor progression (52). Among them, macrophages and dendritic cells often acquire a mixed pro-inflammatory/immune suppressive phenotype both in the mouse (53, 54) and in humans (55, 56). Consequently, emphasis has been placed to identify common mechanisms driving the acquisition of tumor-promoting properties by macrophages and dendritic cells (54, 57–61).

Tumor infiltrating myeloid cells produce tumorigenic, pro-inflammatory cytokines (IL-6, IL-23, TNFα and IL-1) but, oddly enough, also anti-inflammatory cytokines (IL-10, TGFβ) as well as molecules with immune suppressive functions (Arginase1, VEGF, peroxinitrite and Indoleamine 2-3 dioxygenase) (62). What tumor-derived cues might drive this phenotype in tumor-infiltrating myeloid cells is still poorly understood. Even more perplexing is the apparent paradox that the tumor microenvironment is simultaneously pro-inflammatory and immune suppressive for which a mechanistic explanation would be of paramount relevance. As tumor-derived factors promote the transcriptional activation of pro-inflammatory cytokines in myeloid cells (63), then it becomes reasonable to ask whether tumor derived factors are main contributors to the paradox and what triggers them?

In 2011 we reported that cancer cells of various origin undergoing a UPR release a diffusible factor(s) that transmits ER stress to receiver myeloid cells, macrophages and dendritic cells, triggering the secretion of pro-inflammatory/tumorigenic cytokines and the immune suppressive factor Arginase 1 (Arg1) (64, 65). Similarly, others showed that myeloid cells upregulate and secrete VEGF (66). The results and implications of these initial findings have been discussed in several review articles (35, 67, 68) and led to the proposal that a cell-nonautonomous mechanism of intercellular communication at the tumor/myeloid cell interface drives the polarization of myeloid cells in the TME.

While tumor-promoting monocytes/macrophages in renal cell carcinoma and head and neck patients have been shown to possess a mixed pro-inflammmatory/immune suppressive phenotype (55, 69), the role of the UPR in polarizing tumor myeloid cells to this phenotype has indirect support in a series of reports. The transition of macrophages from an anti-tumorigenic to a pro-tumorigenic phenotype was shown to follow signals emanating from the liver TME, and depend on c-JUN phosphorylation in a cell-nonautonomous manner (58) implicating IRE1α, which is known to target the c-JUN N-terminal kinase through TRAF2-ASK1 signaling (70). Murine myeloid suppressor cells are also linked to the UPR in a cell-nonautonomous way through TRAIL receptors (71), suggesting that Arg1-mediated immune suppression may be UPR-initiated (72). Monocytes infiltrating renal cell carcinoma have a distinct transcriptome profile, which includes the upregulation of IRE1α (55). Finally, the constitutive activation of XBP1 in tumor-associated DC drives ovarian cancer progression, whereas DC-specific XBP1 deletion restores their immunostimulatory activity, hence enabling anti-tumor T cell responses (73).

### The IRE1α−XBP1 Pathway Controls the Phenotype of Tumor -Infiltrating Macrophages

To elucidate the mechanism(s) through which the UPR affects immune cells leading to a perturbation of the TME and ultimately immune evasion we studied macrophage polarization using (a) pharmacological inhibitors of the IRE1α and PERK pathway and (b) mice engineered to allow macrophage-specific conditional knock-out of the IRE1α-XBP1 axis (74). CD11b^+/^Gr1^-^ macrophages are polarized to the pro-inflammatory/immune suppressive phenotype during the course of tumor growth in the mouse, hence representing a logical target to interrogate the role of the UPR and its branches. These studies confirmed the IRE1α-dependence of cell-nonautonomous macrophage polarization and ruled out an involvement of the PERK pathway. Also, IRE1α but not PERK inhibition diminished the surface expression of CD86 and PD-L1 suggesting that IRE1α regulates the polarization of macrophages to a pro-inflammatory/immune suppressive phenotype (*Il23p19*, *Il6* and *Arg1*). These results were consistent with reports showing that XBP1 is not only required for the development and survival of bone marrow derived DC (75), but also impedes antigen presentation by lymphoid DC (76, 77) and tumor-associated DC (73). Relevantly, using *Ern1* (the gene coding for IRE1α) or *Xbp1* macrophage conditional knockout mice, we determined that IRE1α-XBP1 axis deficiency in macrophages not only attenuates the development of the pro-inflammatory/immune suppressive phenotype *in vivo* but also reduces PD-L1 expression, significantly slowing growth of B16.F10 melanoma cells *in vivo*, and improving survival of *Ern1* (-/-) over *Ern1 fl/fl* control mice (74). Notably, tumor-infiltrating macrophages in tumor-bearing *Ern1* (-/-) mice had a marked reduction in spliced *Xbp1*, *Il-23p19*, *Arg1 and Cd274* gene expression compared to their *Ern1 fl/fl* counterpart. Thus, in tumor-infiltrating macrophages Ire1α is a key negative regulator of tumor microenvironment immunodynamics, ultimately facilitating tumor growth *in vivo*.

Gene expression analysis using *Ern1(-/-)* or *Xbp1*(-/-) macrophages showed, in addition, that surface PD-L1 expression is subject to two-level control by the IRE1α-XBP1 axis with XBP1-mediated regulation of PD-L1 occurring at the post-translation level, whereas IRE1α-mediated regulation is a transcriptional event. *Ern1*-mediated effects were most likely dependent on RIDD function. Consistently, when we analyzed RNA-Seq data generated from tumor-infiltrating macrophages isolated from thirteen patients with either endometrial or breast cancer we found a strong correlation between *ERN1* and *EIF2AK3* (the gene coding for PERK) (correlation coefficient 0.738; p < 0.003), indicative of UPR activation (74). Since IRE1α function is a multistep and complex process (28) not necessarily captured only by *ERN1* expression levels, we derived a systemic representation of pathway activity controlled by IRE1α and by comparison by PERK. In this model, the IRE1α score predicted *CD274* expression (p-value = 0.040), while the PERK score was non-significant (p-value = 0.103), suggesting that activation of *CD274* gene expression in tumor-infiltrating macrophages depends primarily on the IRE1α pathway.

### UPR Regulation of Myeloid Cells Dysregulates T Cells

It is known that once polarized to a tumor-promoting phenotype (78, 79) myeloid cells restrain anti-tumor T cell responses (80–84). Concomitant events include the secretion of tumor-promoting pro-inflammatory cytokines (IL-6, TNFα and IL23) (63, 85) and the production of immune suppressive factors (e.g., Arg1, TGFβ, and IDO). This orchestra of factors and cell interactions synergize with both CD4 (86–88) and CD8 (89–92) T cells, enhancing their regulatory/suppressive function and contributing to a negative regulation of competent host anti-tumor T cells.

Upon transcellular ER stress conditions, bone marrow-derived DC undergo reduced cell surface display of a high affinity OVA peptide/MHC I complex, but become severely defective in their ability to cross-prime naïve CD8 T cells transgenic for a TCR specific for OVA resulting in significant lack of clonal expansion (65). This proliferative defect is not restored by exogenous IL-2 arguing against typical T cell anergy. Defective cross-priming of CD8 T cells also caused protracted XBP1s upregulation, and CD28 downregulation combined with Foxp3 upregulation, evoking the phenotype of tumor-infiltrating CD8 Tregs in humans (93–96). In line with these finding and the role of the UPR in these regulatory phenomena is the demonstration that abrogation of Xbp1 signaling improves both the antigen-presentation capacity of tumor infiltrating DC and the expansion of naïve CD8 T cells (73).

### NK Cells

The tumor-immune interface comprises other cell types. Among them are natural killer (NK) cells, a specialized population of innate lymphoid cells that mediates cytotoxic functions against damaged, malignant, or virus infected cells (97). Notably, it is estimated that worldwide viruses are linked to ~ 20% of cancers in humans (American Cancer Society). Beside killing target cells NK cells may exert regulatory functions, e.g., facilitating the differentiation of CD4 T cells into Th1 cells, to enhance anti-tumor responses. A general view posits that NK cells discriminate between ‘‘normal and altered self’’ through MHC class I-specific inhibitory receptors and several activating receptors that recognize ligands associated with cell stress. However, an inflammatory tumor microenvironment and tumor-associated cells (macrophages, fibroblasts, myeloid-derived suppressor cells, and Tregs) can decrease or suppress NK cell function (98–100). Genotoxic stress signals activate NK cells *via* upregulation of NKG2D ligands in the mouse (101, 102) and in humans (103). While much is known about the influence of stress on upregulation of NKG2D ligands, little is known on how the UPR regulates the expression of NKG2D in NK. In patients with Type 2 diabetes, a disease characterized by UPR induction in a highly secretory cell environment, NKG2D was found to be markedly decreased compared to normal controls (104). A hint at cell-nonautonomous regulation of NK cells has been provided recently. Tumor-secreted platelet-derived growth factor (PDGF-DD), a factor promoting cellular proliferation, epithelial-mesenchymal transition, stromal reaction, and angiogenesis, was shown to be a ligand for the human immunoreceptor NKp44 expressed on NK cells (105). Paradoxically, while cancer cell production of PDGF supports tumor growth and stromal reaction in a autocrine manner, it concomitantly induces NKp44 expression in NK cells contributing to tumor control. In this study a direct link with the UPR or genotoxic stress was not investigated.

## Aneuploidy in Human Cancer: UPR From Within

Generally, the UPR is considered a reactive phenomenon to metabolic changes in the extracellular milieu reflecting for example nutrient deprivation (protein and glucose) or decreased O_2_ tension (hypoxia), among others. These metabolic dysregulations are commonly associated with cancer. However, UPR can have endogenous origin. For instance, a mutation in the *Muc2* gene, which codes for mucin 2, the main protein in mucus in intestinal “goblet” cells, causes its accumulation in the endoplasmic reticulum and the UPR with associated inflammation (106). A mutation in the Sec61α1, a translocon involved in the transporting newly synthesized polypeptides into the ER lumen, results in UPR in hepatocytes and in heightened sensitivity to UPR in murine pancreatic β cells leading to diabetes (107). Perhaps, these effects can be interpreted in light of the newly discovered role of the Sec61 translocon in limiting the activation of IRE1α during conditions of stress (108).

**Figure 2:**
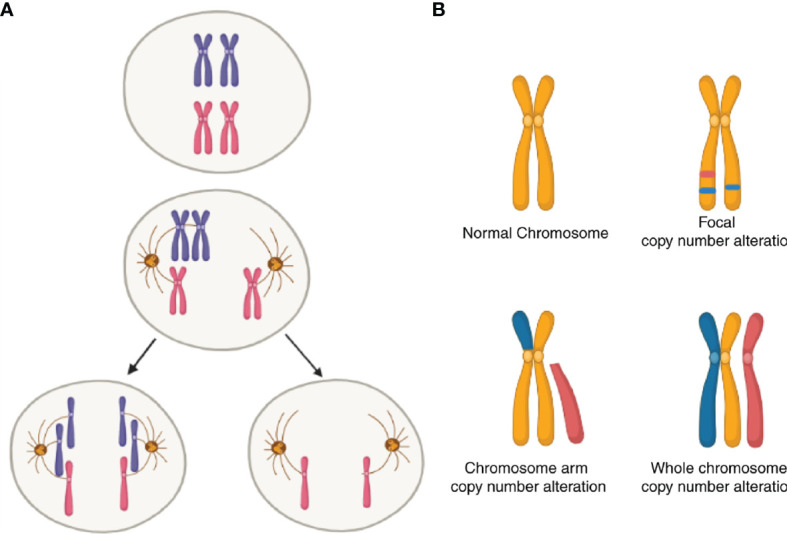
Schematic of somatic copy-number alterations (SCNA). **(A)** Mis-segregation during cell division leads to the production of aneuploidy cells. **(B)** Three broad categories of SCNA, with red color showing regions of copy-number gain and blue color showing regions of copy-number loss. The top-left panel represents a healthy (diploid) chromosome. The top-right panel is a chromosome with *focal* level SCNA events. The bottom-left panel represents *chromosome-arm* level SCNA events. The bottom right panel is *whole-chromosome* level SCNA events.

Another and more general source of UPR from within is aneuploidy. Aneuploidy is the oldest form of chromosomal abnormality identified (109). It can result from mis-segregation during anaphase (e.g., spindle assembly, checkpoint defects; **Figure 2A**) (110), cell fusion (111) or cell-in-cell formation (entosis) (112). In cancer, a broader category of genomic abnormalities called somatic copy-number alterations (SCNA) contribute to tumor cell aneuploidy. Aneuploidy in this context can be divided into three categories: whole-chromosome, chromosome-arm and focal (**Figure 2B**) (113). Aneuploidy is present in 90% of solid tumors and 50% of blood cancers (113, 114). Earlier reports showed that one-quarter of the genome of a typical cancer cell is affected either by *whole-arm* SCNAs or by the *whole-chromosome* SCNAs. *Focal* SCNAs accounts for only 10% of a cancer cell genome (113). Most of whole-chromosome SCNAs preferentially associate with gain or loss (but not both) across cancers (**Figure 2**). However, since aneuploidy is by definition associated with tumor evolution, quantitative estimates are best understood in this dimension. A recent report compared SCNA burden of clonal, i.e., early, with subclonal, i.e., late SCNA and found that 26% of the genome is subject to clonal SCNAs and 18% to subclonal SCNAs. Also, in 45% of tumors, more than 20% of the genome is subject to subclonal SCNAs (115).

About a decade ago, two reports suggested a connection between abnormal ploidy and immune surveillance (116, 117). The two studies, one on breast cancer and the other on colon cancer, showed that the immune response of the host controlled hyperploid neoplastic cells, suggesting that hyperploid cells are subject to “hardwired” immune surveillance. In both studies the proposed mechanism was the upregulation of cell surface expression of calreticulin, an endoplasmic reticulum resident molecule that once translocated to the cell surface serves as an “eat-me” signal for macrophages and dendritic cells. A subsequent independent study suggested that aneuploid cells are selectively eliminated by natural killer (NK) cells (118). However, the claim that NK cells kill aneuploid cells remained unsubstantiated because the IL-2 dependent NK92 cell line, the only NK cell used in the study, yielded no target cell lysis after effector-target cells were incubated for 4 hours, the canonical time of NK assays. Based on these reports one was led to conclude that hyperploid cancer cells incite their selective elimination *via* the initiation of a specific cellular (cytolytic) immune response. Arguably, the studies failed to explain why increased protein content in a hyperploid cancer cell would ostensibly lead to the selective elimination of hyperploid cancer cells (119). If cancer cells are “better” targets for cytotoxic T cells than neighboring non-hyperploid cancer cells, is there immune surveillance of cancer cells without ploidy abnormalities? Beyond the academic aspect of the question, the fact remains that aneuploidy is a progressive process during tumor evolution and is associated with poor prognosis (120–123). Paradoxically, aneuploidy is well tolerated in cancer cells (124–126) and chromosomally unstable cancer cells have increased multidrug resistance (127). Taken together, these facts argue against immune surveillance to selectively eliminate established or emerging aneuploid cells, at least not effectively. Perhaps, cGAS-STING signaling in this context may mediate clearance of aneuploid cells (128).

A different conclusion to the same question was reached by a subsequent study showing that tumor aneuploidy correlates with markers of immune evasion and reduced number of tumor-infiltrating leukocytes (129). This study suggested a connection between aneuploidy and immune surveillance pointing to the potential negative impact aneuploidy plays on local immunity. Does this represent a new important variable in the interplay between cancer and immune cells in the tumor microenvironment?

Several considerations suggest that this may be the case. One is that tumor aneuploidy as a source of genetic variation drives evolutionary selection and advantage (130). The other is that aneuploidy has the potential to trigger the UPR. Studies in yeast and in mammalian cells had previously shown that aneuploidy leads to gene and protein dosage change generating excess client proteins in the endoplasmic reticulum beyond the capacity of quality control/refolding mechanisms (131–133). For instance, in yeast, quantitative changes in the proteome leads to excess misfolded proteins causing the UPR (134). Furthermore, misfolded proteins representing 0.1% of the total proteome are sufficient to elicit the UPR (134). These considerations led to the hypothesis that proteotoxic stress and the UPR in cancer cells could link aneuploidy to dysregulation of immune surveillance (135).

### An Inverse Correlation With Local Immune Cell-Mediated Cytotoxicity

The aforementioned study showed that lower expression level of the immune signature was primarily predicted by high levels of arm and whole-chromosome SCNAs, and that SCNA levels were a stronger predictor of markers of cytotoxic immune cell infiltration than tumor mutational load (129). These conclusions prompted us to develop a standardized and unique aneuploidy score in much the same way a tumor mutational burden (TMB) accounts for the number of non-inherited, nonsynonymous mutations in an individual tumor, an index that is used clinically to assess for example the potential for immune check point blockade (136, 137). Based on this reasoning we first used a pairwise correlation to evaluate the relationship between *whole-chromosome*, *arm* and *focal* SCNA categories and found a positive inter-category correlation (Spearman r= 0.548-0.627) (138). We then derived aggregate scores for each category separately and compared them to a single combined SCNA score and showed that a combined SCNA score had consistently high correlation with all three categories considered independently (Spearman r= 0.735-0.866) with focal SCNA being the least correlated (Spearman r = 0.735). This made it possible to use a single SCNA score (aneuploidy score) to interrogate disease progression and local immune function. A pan-cancer analysis of tumors (n = 6298 and 25 tumor types) for which stage information was available revealed that the single combined SCNA score increased as tumor stage increased with significant positive coefficients (p= 1.39e-09, p= 3.77e-10, p= 2.01e-11) for each tumor stage (138).

**Figure 3:**
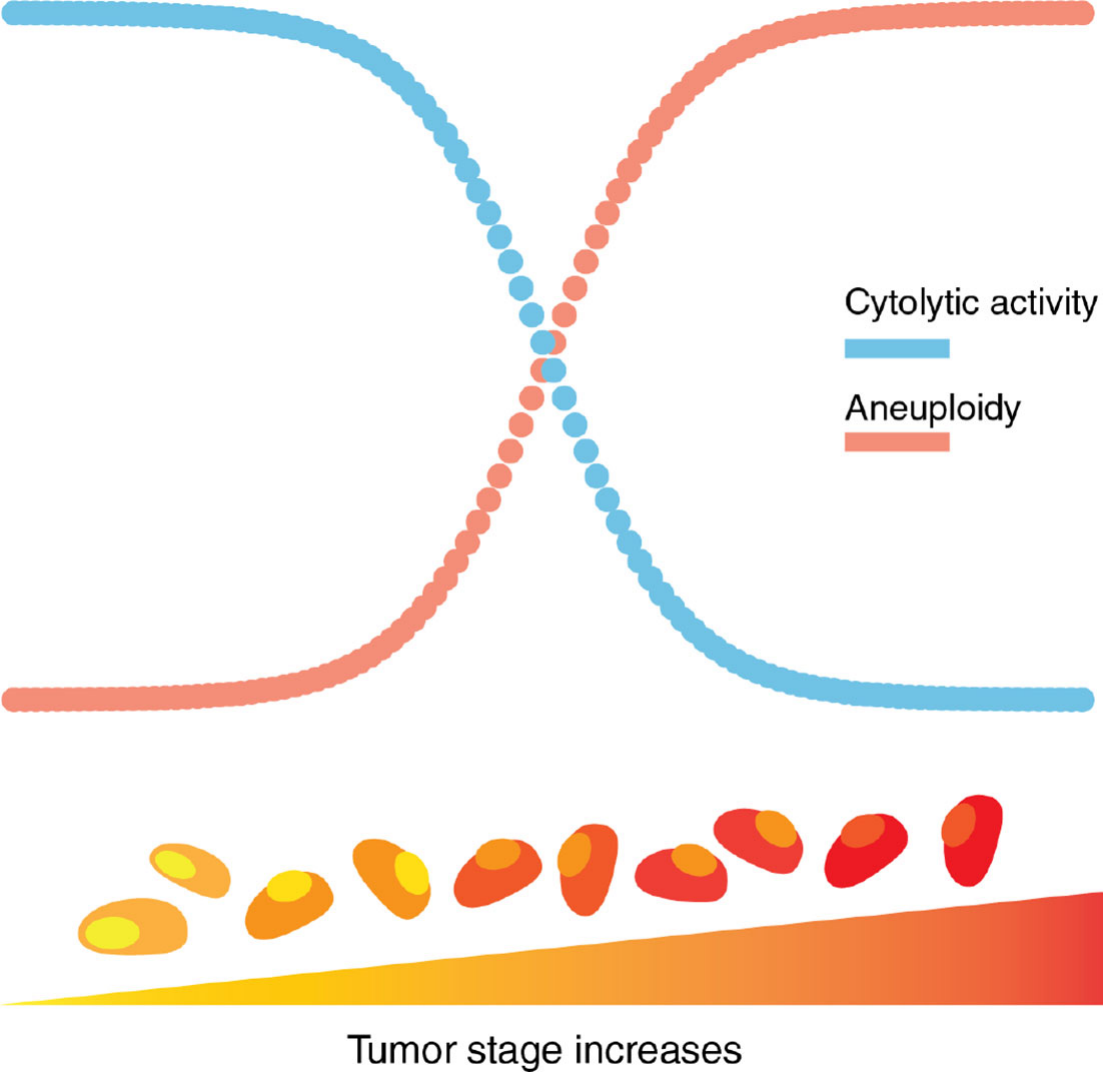
Aneuploidy and cytolytic activity in tumor progression. Aneuploidy, as a measure of chromosomal abnormality, accumulates as tumor stage increases. In contrast, cytolytic activity, a representation of local immune-mediated cytotoxicity, decreases as tumor stage increases.

By measuring perforin (*PRF1*) and granzyme A (*GZMA*) gene expression as representative of cytolytic activity (CYT) in tumors (47) we also found that CYT was inversely correlated with tumor stages across all cancer types. For CYT, we observed almost significant negative coefficients for Stage II (p = 0.075) and a significant negative coefficient for Stage IV (p = 9.74e-5) relative to the levels observed in Stage I tumors. We observed a significant inverse correlation between SCNA score and CYT across all stages pan-cancer, and in most of the tumor types evaluated (138). This finding supports the existence of negative correlation between aneuploidy and immune infiltrate by cells with cytolytic potential (T cells and NK cells). It also shows that as tumors progress SCNA score increases but so does immune evasion (**Figure 3**).

### TCGA Analysis Reveals a Correlation Between UPR Gene Expression and Aneuploidy Score

The UPR is an adaptive survival mechanism used by mammalian cells in response to environmental perturbations, cell-autonomous and cell-nonautonomous, to alleviate the burden of excess client proteins in the ER (27). However, while aneuploid cells are subject to negative selection in healthy tissues usually detrimental to the viability of a healthy cell or organism, it is frequent in cancer and correlates with poor prognosis, suggesting that cancer cells tolerate chromosomal aberrations (126). A potential explanation of this paradox is that telomerase, which is overexpressed in cancer cells, suppresses aneuploidy-induced telomere replication stress, blocking senescence/apoptosis and enabling cell survival (139).

Is there something unique to aneuploidy in cancer cells and what is its relation to the UPR? Of twenty-three tumor types with available matched normal samples in TCGA, all except three (THCA, KICH, and KIRP) showed greater expression of *HSPA5* (the gene coding for GRP78 the master regulator of the UPR) (39), which is also a predictor of resistance to chemotherapy in breast cancer (140). Across all thirty-two tumor types a correlation between the aneuploidy score and parent genes for the three branches of the UPR (IRE1α, PERK and ATF6) was found mainly for three genes from the PERK pathway (*EIF2S1*, *EIF2AK3*, and *DDIT3*), but not for *ATF6* or *ERN1* (the gene coding for IRE1α) or *XBP1*. This primary ER sensor genes analysis suggests that SCNA levels correlate mainly with the PERK pathway. Previous reports showed that PERK is the main branch of the UPR involved in tumor cell adaptation to hypoxic stress in malignant progression, suggesting the importance of translation regulation in these conditions (42). This is also consistent with the observation that transcellular transmission of ER stress between cancer cells is also prevalently regulated by PERK in receiving cells enhancing their survival potential and resilience to chemotherapy (44).

Given that the UPR is a complex signaling system involving many downstream genes, and that aspects of UPR function, IRE1α activity in particular, are regulated by post-translational modifications, we explored the possibility of a correlation between aneuploidy score and downstream effector genes of the three main branches of the UPR, beyond that of parent sensor UPR genes. Over half of the thirty-two tumor types in which an analysis was possible showed significant correlation between SCNA score and the expression of the majority of downstream genes in all three UPR branch pathways. In the same analysis we found that the IRE1α-dependent RIDD activity correlates positively with SCNA and negatively with CYT in several tumor types (138).

**Figure 4:**
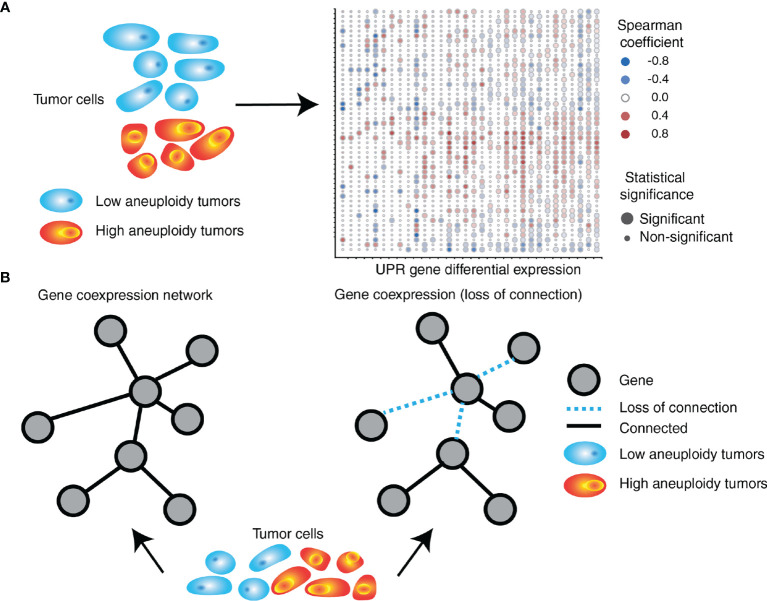
SCNA level associates with perturbation of coordinated UPR gene expression. **(A)** UPR genes are differentially expressed in tumors with high levels of aneuploidy relative to those with low levels of aneuploidy. The right side depicts a heatmap exemplifying gene expression (in rows) differences between high and low aneuploid tumors across 32 different tumor types (in columns) from TCGA. **(B)** UPR gene coordination is altered in high aneuploid tumors relative to low aneuploid tumors. UPR genes in high aneuploid tumors are overall less co-expressed (loss of connection) compared to low aneuploid tumors, with some gene pairs showing unexpected levels of preservation across tumors.

Interestingly, we found a differential co-expression of multiple UPR genes in low (30^th^ percentile) and high (70^th^ percentile) SCNA groups across tumor types. Almost universally we found distinct co-expression patterns of UPR genes, with most tumor types showing less co-expression in the SCNA^high^ compared to the SCNA^low^ group, consistent with general perturbation of the transcriptome by SCNAs (**Figure 4**). UPR branch pathway activities themselves are directly or indirectly affected by SCNAs, and this is related to the magnitude of SCNA (aneuploidy score). This is in line with the principle that in a given process there exists extensive coordination within regulatory levels, e.g., the organization of transcription factors into regulatory motifs, often leading to patterns of gene co-expression (141, 142).

An analysis of consistently differentially co-expressed UPR gene pairs, showed patterns of co-expression changes predominately negative, suggesting loss of coordination (138) (**Figure 4**). Among highly perturbed gene pairs across multiple tumor types are *HSPA5*, *CXXC1*, *SERP1*, *SCH1,* and *PDIA6*, which encode proteins that confer resistance to various forms of stress. On the other hand, co-expression of some gene pairs was preserved across all tumor types despite an increased SCNA score. Ontology analysis of genes with preserved co-expression revealed that these genes are associated with negative regulation of apoptosis. The relationship between *ATF4* and *DDIT3* (the gene coding for CHOP), was often but not always perturbed. In selected instances, *DDIT3* co-expression with *GOSR2* (protein transport) and *ASNS* (asparagine synthetase) remained coordinated, suggesting that some less-known aspects of *DDIT3* activity may benefit tumor cells. For instance, CHOP (the major executioner of apoptosis downstream of irrecoverable UPR) may be required for other functions such as the induction of the proinflammatory/tumorigenic cytokine IL-23 (143).

## Aneuploidy as a Source of Transcellular Effects on Immune Cells

As discussed above a cancer cell UPR promoted by cell-extrinsic noxae is the source of cell-nonautonomous regulation of immune cells. Can aneuploidy, a cell-intrinsic source of UPR do the same? We used two model systems to validate the conclusions of the extensive TCGA analysis. One made use of “quasi-diploid” human cancer cells treated with Reversine (Rv), a small molecule known to induce aneuploidy through inhibition of the mitotic spindle (118, 144). The other utilized clonal murine cell lines resulting from cell-in-cell fusion between B16 melanoma cells and mouse embryonic fibroblasts (MEF) that carry a large number of extra chromosomes (range 72-131) (145). Rv treatment and cell-in-cell fusion both resulted in *XBP1* splicing, a proxy for UPR induction. An analysis of the three branches of the UPR by PCR and Western blotting in human cancer cells treated with Rv showed that aneuploidy triggers a global UPR including the upregulation of GRP78, and an overall activation of both PERK and IRE1α branches, with phosphorylation of eIF2α downstream of PERK being the hallmark of PERK involvement. Thus, two independent models of experimental aneuploidy both pointed to a mechanistic link between aneuploidy and UPR induction consistent with TCGA analysis. Phosphorylation of eIF2α at Ser^51^, a convergent regulatory hub of both the UPR and the integrated stress response (ISR), raised the possibility that eIF2α phosphorylation could be driven by the double-stranded RNA-dependent protein kinase (PKR), the general control non-repressible 2 (GCN2), or the heme-regulated eIF2α kinase (HRI) in addition to PERK (146). All four eIF2α kinases share extensive homology in their kinase catalytic domains, and each responds to distinct environmental and physiological stresses to reflect their unique regulatory mechanisms (146). No other kinase was phosphorylated in Rv-treated cells suggesting that aneuploidy induced by Rv treatment mainly drives eIF2α phosphorylation *via* canonical UPR.

**Figure 5:**
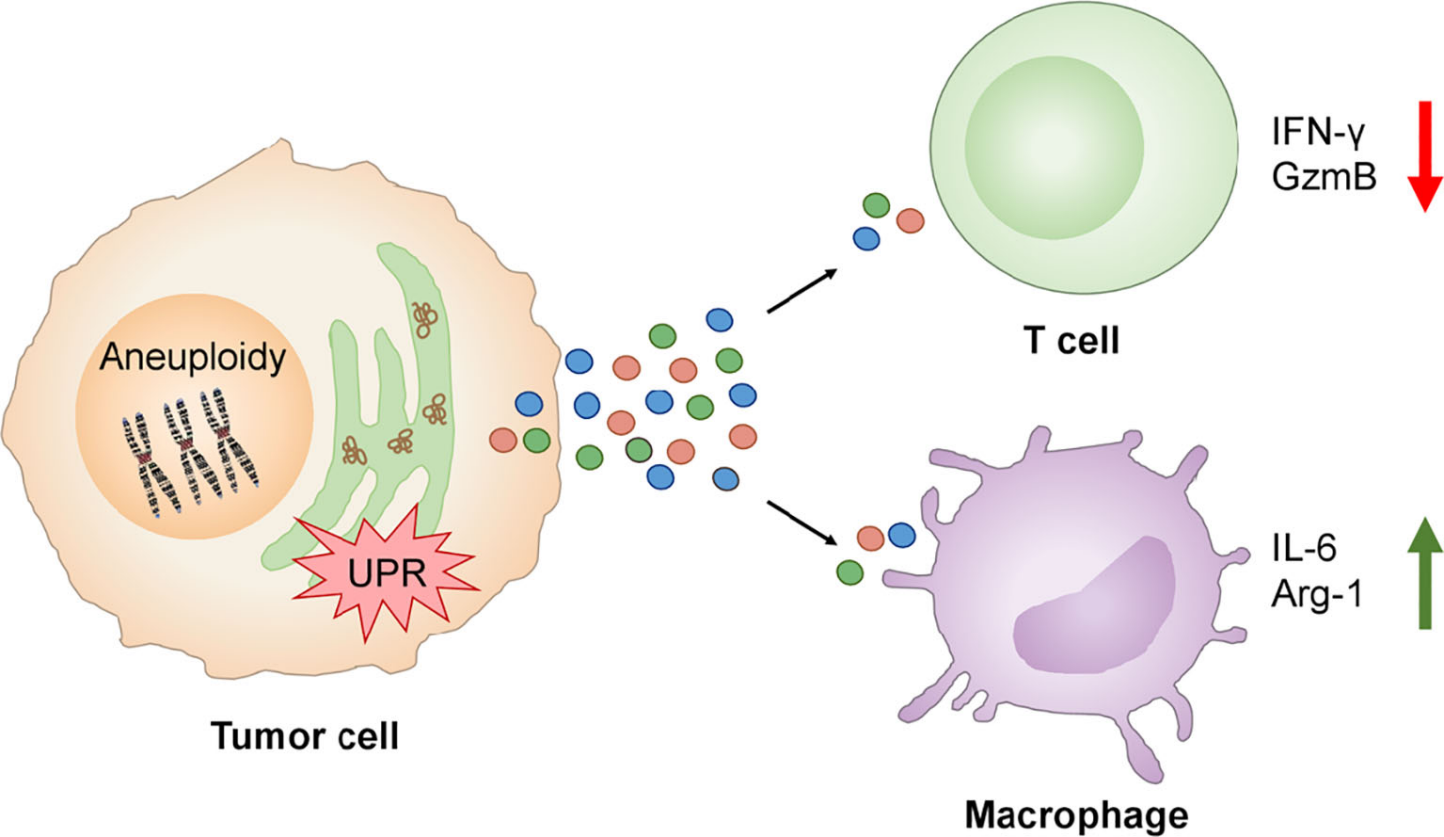
UPR links aneuploidy to immune cell dysregulation in the tumor immune microenvironment. Aneuploidy and UPR activation in tumor cells induces the secretion of factors which inhibit the production of IFN-γ and Granzyme B by activated T cells and promotes expression of IL-6 and Arg-1 by macrophages contributing to local pro-inflammation and immune suppression.

The TCGA analysis showed an inverse correlation between single SCNA score and CYT across disease stages making plausible to ask “Do tumor cells with experimentally-induced aneuploidy also dysregulate T cells through a cell-nonautonomous mechanism?” This possibility was tested focusing on two key functional parameters, IFNγ and Granzyme B, and their production by human T cells activated by anti-CD3/anti-CD28 antibodies. Activation in the presence of conditioned medium from Rv-treated human cancer cells showed marked reduction of both IFNγ (80%) and Granzyme B (60%) relative to control conditioned medium, an effect not due to Rv carry-over. Similarly, conditioned medium of fused B16 cells (aneuploid cells) caused a reduction of IFNγ (55%) and Granzyme B (30%) (138) (**Figure 5**). These results complement those on cross-priming of CD8 T cells by dendritic cells treated with the conditioned medium of ER stressed tumor cells in which defective T cell activation and clonal expansion was observed (65).

The conditioned medium of aneuploid cells collected at the time of maximal *XBP1* splicing also caused *Xbp1* splicing and the expression of *Il6* and *Arg1* in murine bone marrow-derived macrophages. Notably, *Il6* gene expression was induced by the conditioned medium of both Rv-treated human cancer cells or fused murine B16 cells, suggesting that the phenomenon is not species restricted. *Arg1* gene expression was induced by the conditioned medium of Rv-treated human cancer cells only (**Figure 5**). Collectively, these new results suggest that aneuploid cells can affect immune cells in a cell-nonautonomous way, adding aneuploidy as a new player in the dysregulation of local immune cells with the UPR serving as the mechanistic link.

## Conclusions and Perspective

It has been merely a decade since we and others started to characterize the tumor-immune interface from a completely new angle, focusing on the role of the UPR as driver of a dynamic interface that points relentlessly to immune dysregulation. There is little doubt that the tumor-immune interface is a battlefield that is relevant to, and is intricately involved in, tumor progression and immune evasion.

Beyond the cell-autonomous effects of the UPR on cancer cells (33, 34) the influence of cancer cell UPR as a source of cell-nonautonomous effects is more recent and may be considered still in its infancy (35). However, its impact on intercellular communication with macrophages (64), dendritic cells (65, 73), and tumor cells themselves (44) is already evident. In myeloid cells (macrophages and dendritic cells) the effects are directly linked to the production of pro-tumorigenic inflammatory cytokines, immunosuppressive enzymes, and possibly lymphangiogenic factors (147), suggesting that a main function of transcellular UPR is to adapt the phenotype of these cells to a pro-tumorigenic status (67). These cell-nonautonomous effects complement those observed in B cells in which programmed retention of antigen in the endoplasmic reticulum resulted in cell-intrinsic UPR induction with expression of proinflammatory cytokines, negative modulation of the surface expression of MHCII molecules *in vivo*, and decreased expression of OX40L (148), a molecule involved in the generation of memory T cell responses (149–152). The effects of cell-nonautonomous UPR on T cells are equally dramatic, and include inhibition of clonal expansion, reduced activation following cross-priming and reduced tumor infiltration by CD8 T cells (65). Whether or not cell-nonautonomous UPR upregulates PD1 expression on T cells has not been firmly established but the effects of transcellular UPR on T cells are of sufficient relevance to account for local dysregulation of T cell immunity. This needs therefore, to be factored in with other mechanisms of local dysregulation of T cell immunity such as intra-tumor clonal deletion (153), tolerance (154), and exhaustion (155, 156). This emerging scenario should not surprise as examples of cell-nonautonomous signaling through the UPR have been documented in *C. elegans*, increasing longevity and establishing neuroimmune axis communication (157–160).

An additional form of UPR-based, cell-nonautomous immune regulation is immunogenic cell death (ICD) (161). Early mouse experiments testing the anti-tumor effect of classes of chemotherapeutic agents routinely used in the clinic revealed that anthracyclines were unique in causing ICD (162). Today, mouse studies show that anthracyclines, radiotherapy, oncolytic viruses, and photodynamic therapies, can all accentuate the immunogenic potential of dying cancer cells. ICD induction is associated with sustained induction of ROS and ER stress (163). Is ICD a source of transcellular stress at the tumor-immune interface? Anticancer treatments that trigger ICD induce the release (or surface exposure) of immunostimulatory factors or damage-associated molecular patterns (DAMPs). DAMPs immunomodulatory functions behave as a danger signal for immune cells (164). Detailed information on ICD and DAMPs and their relation with the UPR is covered in an recent comprehensive review article (165). DAMPs include the ER chaperone calreticulin (CRT) (“eat me” signal), the heat shock protein (HSP)-70/90 (“eat me” signal), secreted ATP (“find-me” signal), the high mobility group box 1 (HMGB1), a nuclear protein that binds to DNA and activates TLR2 and TLR4 in dendritic cells, and double-stranded DNA that activates endosomal TLR7, TLR8, and TLR9. ICD is linked with PERK signaling (165). However, even though ICD has clear connection with the UPR and triggers the release of immunomodulatory molecules, it is presently unknown if it can also be a source of factors that transmit UPR and pro-inflammation transcellularly. This is relevant since numerous clinical trials are currently testing the ICD paradigm (166). Based on the arguments presented in this review article, cell-nonautonomous UPR as a result of ICD could paradoxically ignite dysregulation of local immunity.

An important new finding is that the UPR links aneuploidy to dysregulation of immune cells. As discussed, aneuploidy is a generator of the UPR from within the cancer cell. Its effects mimic those generated by extrinsic manipulations of the cancer cell UPR (64, 65), spontaneous UPR during tumor growth *in vivo* (74), and genetic manipulation of UPR branches (73, 74). Of paramount relevance aneuploid cells impart changes to activated T cells such as decreased IFNγ and Granzyme B production that are similar to changes reported for CD4 T cells in ascites of ovarian cancer patients (167), or changes in gene expression noted in head & neck and urothelial cancers with an aneuploid switch based on chromosome 9p arm loss or homozygous deletion of 9p21.3 (9p21) (168, 169). Aneuploidy also has the potential to fuel inflammation at the tumor-immune interface through the mechanism illustrated herein. However, aneuploidy is not the only mechanism generating inflammation in the tumor microenvironment, contributing to intratumoral immune dysregulation. In a circular process chronic inflammation could even generate chromosomal instability (170), perhaps through miRNAs that are abundant during inflammation (e.g., miR-155), which was shown to down-regulate WEE1, a kinase that blocks cell-cycle progression and the DNA mismatch repair system (171). It has been postulated that this could lead to loss of heterozygosity (LOH) and aneuploidy (172).

The principle of transcellular communication in cancer is new and contrasts current views on manipulation of the cancer immune response based on precision immunotherapy, e.g., the induction of T cells against neoantigens and immune checkpoint blockade. However, it has also become apparent that there exist numerous hurdles to targeted immunotherapies by neoantigens (173) and immune checkpoint inhibitors (174, 175). A possible explanation is that tumor is far from being a static element. Notwithstanding the impact of genomic evolution, tumors also evolve in symbiosis with their microenvironment. Intercellular communication mechanisms create homogeneity in a mixed cell population in spite of genomic alterations, which remain poor predictors of response to immunotherapy except perhaps for clonal mutational tumor burden (176), which faces the challenge of being progressively ignored by the MHC of the host (177, 178). We believe that cell-nonautonomous regulation likely operates independently of genomic alterations to coordinate community behavior and survival in the natural habitat, leveraging a cooperative behavior among subclones that can influence disease progression (179). In solid tumors cell cooperation generates a *fitter* population of cells as demonstrated by clonal repopulation dynamics experiments where breast cancer cells with identical genetic mutations acquired different clonal behavior *in vivo* and malignant phenotypes contributed by a sub-population of cells stimulated the growth of all other cells (180, 181). It should, therefore, not surprise that cell-nonautonomous regulation of clonal evolution and tumor growth (182) and tumor cell heterogeneity (183) may also be the product of local interactions among cells. Current understanding indicates that UPR-based cell-nonautonomous effects in the TME play a major role in remodeling the function of infiltrating immune cells, dysregulating their protective function while creating an immune suppressive environment. As shown, aneuploidy represents a source of UPR from within that adds an extra layer of complexity to understand and correct immune dysregulation of the tumor-immune interface.

Whereas cancer comprises in excess of 100 different disease entities with diverse risk factors and epidemiology, the new view provides for an evolutionarily conserved mechanism, common to tumors of different tissue origins and irrespective of the initiating mechanism, which appears to be more conserved in human cancers than some common driver mutations (e.g., p53, K-Ras and Pten). From an evolutionary biology standpoint, principles of cell-nonautonomous regulation conform to criteria of selection for cell fitness. In multicellular organisms this requires the suppression of cell-level fitness to the advantage of organism-level fitness (184). Although intercellular communication is the byproduct of mutualism driven by *resource sharing* (20), cancer cells represent the exception and a breakdown of this this principle if one considers that the cell-nonautonomous effects of cancer UPR are not based on *resource sharing* but rather to generate tumors with greater fitness while effectively impeding control by the immune system. We believe that this forms the basis of what can be termed environmental selection.

The gain in knowledge of the past decade has implication for therapy. As discussed, the available data suggest a paradox where a cell-autonomous UPR is primarily driven by the PERK branch whereas immune cells recipient of a transcellular UPR appear to be regulated at the level of the IRE1α branch. Exception to this emerging rule is a report showing that dysregulation of T cells by the UPR is primarily mediate by PERK-eIF2α phosphorylation through attenuation of protein synthesis (185). In our opinion this provides the opportunity to intervene selectively at the cancer or the immune cell level to either regulate cancer cell survival and reverse resistance to therapy, or block immune cell dysregulation to potentiate immunotherapies but also restore autochthonous immunosurveillance. The ideas discussed herein warrant future studies to more precisely understand how manipulation of the UPR in the tumor microenvironment can lead to a greater immunological control of cancer.

## Author Contributions

MZ wrote the article. SX edited and contributed iconographic material. MD edited and contributed iconographic material. HC revised and edited article. All authors contributed to the article and approved the submitted version.

## Funding

This work was supported in part by grant NIH RO1 CA220009 to MZ and HC and a Mark Foundation Emerging Leader Award 18-022-ELA to HC.

## Conflict of Interest

The authors declare that the research was conducted in the absence of any commercial or financial relationships that could be construed as a potential conflict of interest.

## Publisher’s Note

All claims expressed in this article are solely those of the authors and do not necessarily represent those of their affiliated organizations, or those of the publisher, the editors and the reviewers. Any product that may be evaluated in this article, or claim that may be made by its manufacturer, is not guaranteed or endorsed by the publisher.
